# Evaluation of fisetin as a potential inducer of mitochondrial biogenesis in SH-SY5Y neuronal cells

**DOI:** 10.22038/IJBMS.2023.72272.15714

**Published:** 2023

**Authors:** Muhammet Ay

**Affiliations:** Department of Genetics and Bioengineering, Alanya Alaaddin Keykubat University, Alanya, Antalya, Turkey

**Keywords:** Fisetin, Mitochondria, mtDNA, Parkinson’s disease, PCR, SH-SY5Y

## Abstract

**Objective(s):**

Increasing evidence implicates impaired mitochondrial biogenesis as an important contributor to mitochondrial dysfunction, which plays a central role in the pathogenesis of neurodegenerative diseases including Parkinson’s disease (PD). For this reason, targeting mitochondrial biogenesis may present a promising therapeutic strategy for PD. The present study attempted to investigate the effects of fisetin, a dietary flavonoid, on mitochondrial biogenesis and the expression of PD-associated genes in neuronal cells.

**Materials and Methods:**

The effects of fisetin on mitochondrial biogenesis were evaluated by three different approaches; PGC-1α and TFAM mRNA expressions by RT-qPCR, mitochondrial DNA (mtDNA) content by quantitative PCR and mitochondrial mass by MitoTracker staining. Next, a PCR array was performed to evaluate the effects of fisetin on the expression profile of PD-associated genes. Finally, the common targets of fisetin and PD were analyzed by *in silico* analyses.

**Results:**

The results demonstrated that fisetin treatment can increase PGC-1α and TFAM mRNA levels, mtDNA copy number, and mitochondrial mass in neuronal cells. Fisetin also altered the expressions of some PD-related genes involved in neuroprotection and neuronal differentiation. Moreover, the bioinformatics analyses suggested that the AKT1-GSK3B signaling might be responsible for the potential neuroprotective effects of fisetin.

**Conclusion:**

Collectively, these results imply that fisetin could modulate some neuroprotective mechanisms including mitochondrial biogenesis, and may serve as a potential drug candidate for PD.

## Introduction

Parkinson’s disease (PD) is the most common movement disorder afflicting around 6 million people worldwide (1). PD is pathologically characterized by the progressive death of dopaminergic neurons and the Lewy body formation in the substantia nigra and the significant reduction of striatal dopamine (2, 3). Although the molecular mechanisms of dopaminergic neurodegeneration are still largely unknown, mitochondrial dysfunction, neuroinflammation, and oxidative stress have been considered key mechanisms in the pathogenesis of PD (4, 5). Mitochondrial function is crucial for the survival of high energy-demanding cells such as neurons and is maintained by mitochondrial fusion, fission, biogenesis, and mitophagy processes (6, 7). Mitochondrial biogenesis is an elaborated process comprising the growth and division of mitochondria already present and is coordinated by both the mitochondrial and nuclear genomes (8). A growing body of literature indicates that impaired mitochondrial biogenesis can cause mitochondrial dysfunction (9-11). Thus, improving mitochondrial biogenesis by natural compounds may serve as an attractive strategy for preventing and treating PD. 

Fisetin is a dietary flavonoid widely found in various vegetables and fruits such as strawberries, apples, persimmons, onions, grapes, kiwis, and cucumbers (12). Among them, strawberries have been reported to contain the highest concentration of fisetin (160 μg/g) (13). Previous reports have documented the anti-inflammatory, anti-oxidant, and neurotropic properties of fisetin (14, 15) and thus fisetin has been gaining appreciable attention as a potential natural neuroprotective molecule. In recent years, fisetin has been demonstrated to exert protective effects in both *in vitro* and *in vivo* studies of PD. Fisetin has been reported to alleviate 6-OHDA-, MPP^+^-, and rotenone-induced neurotoxicity in cell culture models of PD (16-18). Fisetin has also been demonstrated to diminish α-synuclein aggregation and α-synuclein-induced toxicity in yeast cells (19). More importantly, fisetin was effective in protecting against behavioral deficits and dopaminergic neurodegeneration in MPTP-treated mice (20) and in reversing biochemical and behavioral impairments in rotenone-treated rats (21). However, the molecular mechanisms underlying the anti-parkinsonian effects of fisetin have not been well clarified. This study hypothesized that fisetin can act as an antiparkinsonian agent through increasing mitochondrial biogenesis and thus examined the effects of fisetin on mitochondrial biogenesis and on the expression of PD-associated genes in neuronal cells.

## Materials and Methods

***Reagents***

Dulbecco’s modified Eagle’s medium (DMEM), L-glutamine, penicillin-streptomycin, fetal bovine serum (FBS), trypsin-EDTA, dimethyl sulfoxide (DMSO), fisetin, and thiazolyl blue tetrazolium bromide (MTT) were obtained from Sigma-Aldrich (St. Louis, MO, USA). High-Capacity cDNA Reverse Transcription Kit, and MitoTracker™ Green FM were purchased from Thermo Fisher Scientific (Waltham, MA, USA). Absolutely RNA Miniprep Kit was obtained from Agilent Technologies (Santa Clara, CA, USA). TFAM, PGC-1α and GAPDH QuantiTect primer assays, RT² Profiler™ PCR Array Human Parkinson’s Disease, RT² SYBR Green qPCR Mastermix, and DNeasy Blood & Tissue Kit were purchased from Qiagen (Valencia, CA, USA). 

***Cell culture***

SH-SY5Y cells were grown in DMEM (Sigma-Aldrich) containing 10% fetal bovine serum (FBS; Sigma-Aldrich), 2 mM L-glutamine, 50 μg/ml streptomycin, and 50 units penicillin and maintained at 37 °C in a 5% CO_2_ atmosphere. SH-SY5Y cells were cultured in T25 flasks or multi-well plates and the medium was changed three times a week. 

***Cell viability test***

The colorimetric MTT test was performed, as described previously (22). Briefly, the cells (1×10^4^ cells/well) were plated in 96-well plates. The next day, cells were incubated with varying concentrations of fisetin for 24 hr. After the treatment, the cells were incubated with MTT solution for 3–4 hr at 37 °C. Following the incubation with MTT, the supernatants were carefully removed and the formazan crystals were dissolved in DMSO. The absorbance was read at 570 nm using a microplate reader (BioTek Instruments, Winooski, VT, USA). 

***RT-qPCR***

Total RNA was extracted from SH-SY5Y cells using the Absolutely RNA Miniprep kit (Agilent Technologies, Santa Clara, CA, USA). After measuring RNA concentrations, 1 μg RNA was converted to cDNA using the High Capacity cDNA Reverse Transcription kit (Thermo Fisher Scientific, Waltham, MA, USA). RT-qPCR reactions were carried out on a LightCycler 96 (Roche Diagnostics) using the RT^2^ SYBR Green qPCR Mastermix kit (Qiagen) and QuantiTect Primer Assay kits (Qiagen). All reactions were performed in triplicate in a final volume of 20 µl. Data were analyzed using the comparative threshold cycle (Ct) method and normalized to the GAPDH reference gene.

***Analysis of mtDNA copy number***

SH-SY5Y neuronal cells (1x10^6^ cells/well) were seeded in T25 flasks. After overnight culture, cells were incubated with 10 µM fisetin for 24 hr. Following treatment, genomic DNA was obtained from fisetin-treated cells using the DNeasy Blood and Tissue kit (Qiagen, Valencia, CA, USA). Relative copy numbers of mtDNA were determined by quantitative real-time PCR. Briefly, 10 ng DNA was amplified using the primers for mitochondrial ND1 and nuclear GAPDH genes and the reactions were carried out using a LightCycler 96 instrument (Roche Diagnostics). The relative mtDNA copy number was calculated by a comparative Ct method. 

***Mitotracker assay***

SH-SY5Y cells were seeded into 96-well plates at a density of 1 × 10^4^ cells/well. Twenty-four hours later, cells were exposed to 10 µM fisetin for 24 hr. At the end of treatment, cells were washed with serum-free medium and incubated with 200 nM MitoTracker Green FM (Invitrogen) for 30 min. After staining the cells, cells were washed with PBS three times, and then fluorescence was read on a fluorescence microplate reader (excitation 485 nm, emission 520 nm). 

***RT² profiler PCR array***

The Human Parkinson’s Disease PCR array (Qiagen, PAHS-124Z) was employed to determine the expressions of 84 genes associated with PD. RNA extraction and cDNA synthesis were performed as mentioned in *RT-qPCR*. The cDNA was mixed with the RT^2^ SYBR Green qPCR mastermix and RT-qPCR was carried out using a LightCycler 96 instrument. The data were normalized using the average expression of five housekeeping genes (β-actin, ACTB; β2-microglobulin, B2M; glyceraldehyde-3-phosphate dehydrogenase, GAPDH; hypoxanthine phosphoribosyltransferase 1, HPRT1; ribosomal protein large P0, RPLP0). The expression fold change or regulation was calculated through the RT^2^ Profiler PCR Array Data Analysis Webportal using the comparative Ct method. 

***Bioinformatic analyses***

The SMILES code of fisetin was obtained from PubChem (https://pubchem.ncbi.nlm.nih.gov/) and entered into the SwissTargetPrediction server (http://www.swisstargetprediction.ch/) to identify the potential target proteins (23). The targets with a probability value >0.5 were selected. Next, PD-related targets were obtained from the GeneCards database (https://www.genecards.org/), and the top 500 of total 7984 targets were retained (24). The overlapping targets of fisetin and PD were visualized using a Venn diagram (https://bioinformatics.psb.ugent.be/webtools/Venn/). The fourteen common targets of fisetin and PD were uploaded to the STRING database (https://string-db.org/) to build a protein-protein interaction (PPI) network (25). 

***Statistical analysis***

Statistical analyses were performed using the GraphPad Prism 5 software. The control and treatment groups were compared using the Student’s *t*-test and the differences with *P*<0.05 were considered significant.

## Results

***Cytotoxicity of fisetin in SH-SY5Y cells***

**Figure 1:**
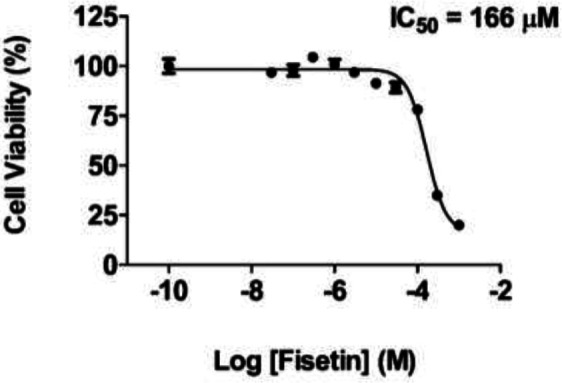
Cytotoxicity of fisetin in SH-SY5Y cells

First, the cytotoxicity of fisetin was assessed in SH-SY5Y cells to find out the IC_50_ value and an optimal dose of fisetin. After treating the cells with different concentrations of fisetin (30 nM–1 mM) for 24 hr, cell viability was assessed using the MTT assay. From the MTT assay measurements, the IC50 value of fisetin was calculated as 166 µM, and 10 µM was chosen as the optimal dose of fisetin for further experiments (Figure 1). 

***Fisetin increases mitochondrial biogenesis in SH-SY5Y neuronal cells***

**Figure 2:**
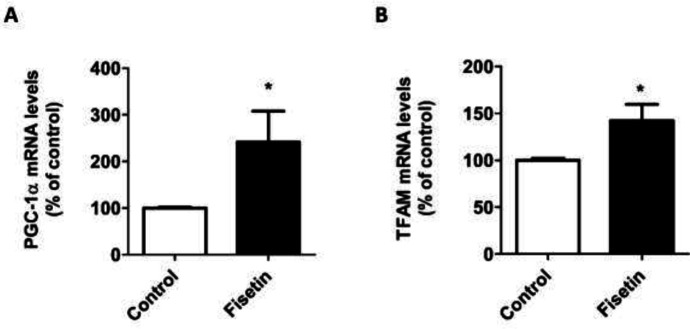
Effect of fisetin on PGC-1α and TFAM gene expression in SH-SY5Y cells

**Figure 3:**
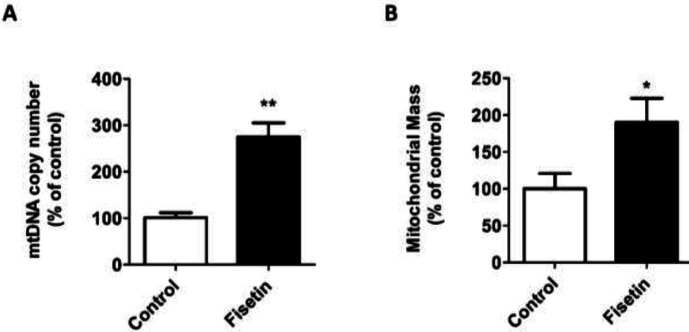
Effect of fisetin on mtDNA copy number and mitochondrial mass in SH-SY5Y cells

After determining the optimal dose of fisetin, mitochondrial biogenesis was assessed in fisetin-treated SH-SY5Y cells. Given that PGC-1α and TFAM are the major genes controlling mitochondrial biogenesis (26), the expressions of PGC-1α and TFAM genes were measured in fisetin-treated cells. Exposure of SH-SY5Y cells to 10 µM fisetin for 18 hr resulted in significant increases in PGC-1α (Figure 2A) and TFAM (Figure 2B) mRNA levels. To substantiate the positive effects of fisetin on mitochondrial biogenesis, the mtDNA copy number, and mitochondrial mass were also evaluated in fisetin-treated cells. As shown in Figure 3A, the levels of mtDNA copy number were significantly elevated in SH-SY5Y cells treated with fisetin. Moreover, MitoTracker staining assay revealed that fisetin-treated cells possessed higher mitochondrial mass than vehicle-treated cells (Figure 3B). Taken together, these results suggest that fisetin is able to increase mitochondrial biogenesis in SH-SY5Y cells.

***Fisetin alters the expression of some PD-associated genes***

**Figure 4:**
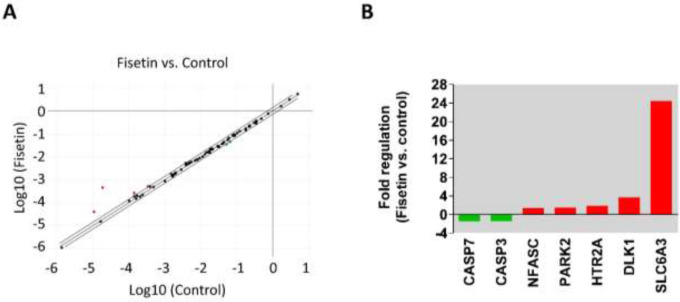
Effect of fisetin on the expression of PD-related genes

To explore the potential of fisetin as an anti-parkinsonian molecule, the mRNA expressions of PD-associated genes were measured in fisetin-treated cells by using the RT² Profiler PCR array. The mRNA levels of 84 PD-related genes were assessed in SH-SY5Y neuronal cells exposed to 10 µM fisetin for 24 hr. The results revealed that the expressions of 7 genes were altered at least 1.4 fold in SH-SY5Y cells treated with fisetin (Figure 4A). Among the differentially expressed genes, the apoptotic genes caspase-3 (CASP3) and caspase-7 (CASP7) were down-regulated in fisetin-treated cells (Figure 4B). Besides, the SLC6A3 was found to be the highest up-regulated gene, followed by the DLK1, HTR2A, PARK2, and NFASC genes (Figure 4B). 

***In silico*** ***analyses of the common targets of fisetin and PD***

**Figure 5:**
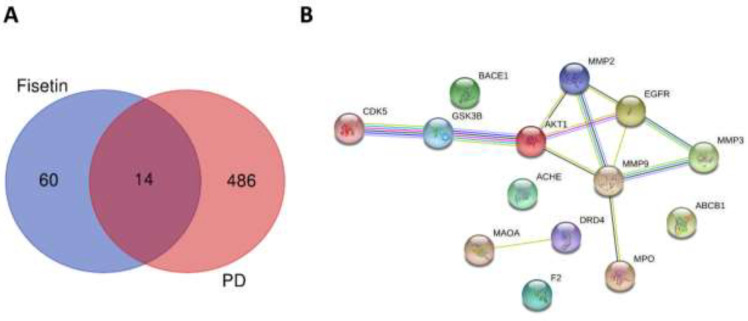
Identification and STRING analysis of the common targets of fisetin and PD

To better understand the therapeutic potential of fisetin in PD, an *in silico * analysis was performed to identify the common targets of fisetin and PD and to construct a PPI network of overlapping targets. The SwissTargetPrediction database was used to identify the targets of fisetin and the targets with a probability value >0.5 were chosen as potential targets of fisetin. The PD-related targets were predicted using the GeneCards database and the top 500 of a total of 7984 targets were selected. Then, the common targets of fisetin and PD were obtained by Venn diagram (Figure 5A). After identifying the shared targets of fisetin and PD, the PPI network of these 14 common targets was built using the STRING software with a high confidence level (0.700) (Figure 5B). STRING analysis demonstrated that GSK3B has strong interactions with AKT1 and CDK5. Moreover, the results also showed that MMP9 is closely associated with MMP2 and MMP3. 

MTT assay was performed to assess the viability of SH-SY5Y cells treated with fisetin (30 nM–1 mM) for 24 hr. Data are presented as the mean±SEM of n = 6 biological replicates from two independent experiments.

SH-SY5Y cells were incubated with 10 µM fisetin for 18 hr and gene expressions of PGC-1α (A) and TFAM (B) were measured by RT-qPCR. GAPDH was used as the housekeeping gene. Data were analyzed using the Student's t-test (*, *P*<0.05 comparing control and fisetin-treated samples). Data are presented as the mean±SEM of three independent experiments performed in duplicate.

A) qPCR was performed to assess the mtDNA content of SH-SY5Y cells treated with vehicle or 10 µM fisetin for 24 hr (n=6). Data were analyzed using the Student's t-test (**, *P*<0.01). Data are presented as the mean±SEM of two independent experiments performed in triplicate. B) MitoTracker staining was performed in SH-SY5Y cells treated with vehicle or 10 µM fisetin for 24 hr (n=6). Data were analyzed using the Student's t-test (*, *P*<0.05). Data are presented as the mean±SEM of two independent experiments performed in triplicate.

A and B, the mRNA expressions of PD-associated genes were determined using RT² Profiler PCR array. A) Scatter plot of PCR array results. Red dots indicate up-regulated genes and green dots indicate down-regulated genes. The central lines indicate genes whose expression was changed by ≤ 1.4-fold. B) The graph shows the up-regulated and down-regulated genes by at least 1.4-fold. PD: Parkinson’s disease

A) Venn diagram of the common targets of fisetin and PD. B) STRING analysis of the 14 overlapping targets of fisetin and PD.

PD: Parkinson’s disease

## Discussion

PD is considered a multifactorial disease, resulting from a variety of genetic and environmental factors (27). The complex interactions between genetic and environmental factors may contribute to several pathophysiological events of PD, including oxidative stress, mitochondrial dysfunction, and neuroinflammation (28). Because of the multifaceted nature of PD, natural compounds that possess the ability to modulate multiple biological pathways have been gaining considerable interest as potential antiparkinsonian agents. Fisetin, a phenolic compound primarily found in strawberries, has been evaluated in some previous studies of PD and found to display protective effects by reducing oxidative stress, neuroinflammation, and alpha-synuclein aggregation (17-19). Even though a recent report has shown that fisetin can alleviate mitochondrial dysfunction by increasing the levels of mitochondrial enzymes in an animal model of PD (21), the effects of fisetin on mitochondrial mechanisms remain mostly elusive. This study aimed to investigate how fisetin affects mitochondrial biogenesis and the expression of PD-associated genes in neuronal cells and to identify the common molecular targets of fisetin and PD using *in silico * analyses. The results demonstrate that fisetin can enhance mitochondrial biogenesis and change the expression of some PD-associated genes in SH-SY5Y neuronal cells and that fisetin and PD share some molecular targets which are involved in mitochondrial pathways. 

Impaired mitochondrial function has been strongly implicated in the pathogenesis of PD (29, 30). Therefore, improving mitochondrial function via targeting a mitochondrial process such as biogenesis, mitophagy, or dynamics has been investigated as a neuroprotective strategy for PD, and various natural molecules that can enhance mitochondrial activity have displayed neuroprotective effects against dopaminergic neurodegeneration (31-33). In the present study, the effect of fisetin on mitochondrial biogenesis was assessed in neuronal cells. Although fisetin has previously been demonstrated to have neuroprotective effects in *C. elegans* through its mitochondrial uncoupling activity (34) and to elevate the levels of some mitochondrial enzymes in rotenone-treated rat brains (21), the effects of fisetin on mitochondrial biogenesis still remain mostly unknown. The findings of this study showed that treatment of cells with a non-toxic dose of fisetin caused significant elevations in the mRNA levels of PGC-1α and TFAM, which are well-known regulators of mitochondrial biogenesis. Importantly, increased levels of mtDNA copy number and mitochondrial mass were also observed in fisetin-treated cells. The number of mtDNA copies has been considered as a good indicator of mitochondrial function and biogenesis and was found lower in the blood and brain samples of PD patients compared to controls (35, 36). The current results suggest that fisetin can serve as a positive modulator of mitochondrial biogenesis in neuronal cells and is thus worthwhile to study as a potential neuroprotective agent.

To assess fisetin’s potential as an antiparkinsonian agent, a PCR array was performed to determine the expression profile of 84 genes, which are directly or indirectly associated with PD. PCR array data demonstrated that fisetin treatment affected the mRNA expression of 7 PD-associated genes by more than 1.4-fold. Interestingly, exposure of cells to fisetin gave rise to the down-regulation of basal caspase-3 and caspase-7 expressions. Previously, fisetin has been shown to attenuate 6-OHDA-induced caspase-3/7 activation (18) and spinal cord injury-induced caspase-3 mRNA expression (37). In line with previously published data, these results suggest that fisetin can even reduce basal caspase activation in neuronal cells. On the other hand, the findings of the PCR array showed that fisetin elevated the SLC6A3 mRNA expression approximately 24-fold. The SLC6A3 gene encodes a dopamine transporter (DAT) and is one of the marker genes of dopaminergic neurons (38). The second most up-regulated gene in fisetin-treated neuronal cells was DLK1, which plays a role in dopaminergic neuron differentiation (39). Among the other up-regulated genes, HTR2A and PARK2 have previously been demonstrated to regulate mitochondrial biogenesis in neurons (40, 41) raising the possibility that fisetin may increase mitochondrial biogenesis via up-regulation of HTR2A and PARK2. Finally, neurofascin (NFASC) mRNA expression was also found to be elevated in cells treated with fisetin. NFASC is an axonal protein that plays an important role in the maintenance of axonal integrity (42). Interestingly, overexpression of NFASC has been shown to protect against mutant α-synuclein-induced neurotoxicity (43). Collectively, these findings imply that fisetin has the potential to up-regulate some genes associated with neuronal differentiation, mitochondrial biogenesis, and neuroprotection, suggesting a protective role of fisetin in neuronal cells.

In order to investigate the possible molecular mechanisms of action of fisetin in PD, bioinformatic analysis was performed. The common targets of fisetin and PD were identified and a PPI network of the 14 shared targets was built using the STRING database. STRING analysis showed that GSK3B has been associated with CDK5 with a high confidence score (0.955) and that there is a strong relationship between GSK3B and AKT1 with a confidence score of 0.996. It is noteworthy that AKT1 signaling has long been known to negatively regulate the GSK3B activity (44-46). Fisetin has previously been reported to inhibit GSK3B (47), which can inhibit mitochondrial biogenesis. Moreover, it has been reported that inhibition of GSK3B protects against dopaminergic neurodegeneration (48) and also increases brain energy metabolism (49). Fisetin has also been shown to activate the AKT1 signaling in an *in vitro* model of PD (13) and amyloid beta (Aβ)-treated mice (50). AKT1 has been reported to phosphorylate and increase the nuclear translocation of NRF-1, which can regulate mitochondrial biogenesis through TFAM expression (51). In addition, inhibition of AKT1 activation has been demonstrated to decrease PGC-1α expression in a rat model of AD (52), suggesting a regulatory role of AKT1 in mitochondrial biogenesis. Given that small molecule inhibitors of GSK3B and activators of AKT1 have been reported to possess protective effects in experimental models of PD (48, 53), fisetin could be a promising neuroprotective agent for PD due to its ability to inhibit GSK3B or activate AKT1. 

There are some limitations in this study. The primary limitation is the *in vitro* nature of the study and these results should be confirmed in an *in vivo* study. Secondly, the PCR array results should be verified by individual RT-qPCR experiments. Thirdly, the findings of *in silico * analyses need to be confirmed experimentally for more accurate identification of fisetin targets. Lastly, the positive effects of fisetin on mitochondrial biogenesis can be further evaluated by Seahorse mitochondrial bioenergetic analysis. 

## Conclusion

These results provide evidence that fisetin can increase mitochondrial biogenesis in SH-SY5Y neuronal cells. In addition to enhancing mitochondrial biogenesis, fisetin can also regulate the expressions of some genes associated with neuronal differentiation and neuroprotection as evidenced by the PCR array experiment. Moreover, *in silico * analyses revealed that AKT1-GSK3B signaling could play a role in governing the protective effects of fisetin in neuronal cells. Finally, the results obtained from these *in vitro* and *in silico * studies should be investigated thoroughly in animal models of PD to substantiate the neuroprotective potential of fisetin.

## Authors’ Contributions

M A designed and performed the experiments, collected data, discussed the results, directed and managed the study, wrote the manuscript and acquired funding.

## Conflicts of Interest

None.
